# Computational Enzyme Design: Advances, hurdles and possible ways forward

**DOI:** 10.5936/csbj.201209009

**Published:** 2012-10-23

**Authors:** Mats Linder

**Affiliations:** a Applied Physical Chemistry, KTH Royal Institute of Technology, Teknikringen 30, SE-100 44, Stockholm, Sweden

## Abstract

This mini review addresses recent developments in computational enzyme design. Successful protocols as well as known issues and limitations are discussed from an energetic perspective. It will be argued that improved results can be obtained by including a dynamic treatment in the design protocol. Finally, a molecular dynamics-based approach for evaluating and refining computational designs is presented.

## 1. Introduction

A key factor in the success of the human race is our inclination to exploit nature for our own ends. Even though it has been disastrous many times throughout history, we would be nowhere near the current height of our civilization the without this trait.

One frontier waiting to be conquered is the control over enzyme catalysis. Enzymes have intrigued us for as long as they have been known because of their formidable complexity and proficiency, stemming from a relatively low number of catalyzed chemical transformations.[1] It is conceived that if we can take control of these 'unit operations’ and their structure-function relationships, we will be able to design environmentally benign catalysts, yet featuring superior efficiency and selectivity compared to those in use today.[2] Another, more fundamental aspect is that the successful design of an enzyme is a stringent demonstration that the underlying mechanics have been understood. Given the increasing interest for industrial biocatalysis and the need for highly specific solutions to synthetic problems, the expectations on artificial (or *de novo*) enzymes for the coming decades are high.[2]

Recent years have seen tremendous advancement in computer-aided (or computational) enzyme design. The field can be defined as the use of *in silico* methods to understand, model and enhance/construct enzyme catalysis. As with all computational disciplines, the limits for what can be done are continuously pushed forward, from early approaches with limited side-chain rotamer optimization,[3–8] to more sophisticated optimization algorithms available today.[9–14] In addition, highly interesting crowd-sourcing applications have been applied in recent years, such as Rosetta@Home[15] and FoldIt.[16, 17]

A long-standing vision in computational protein design has been to *automate*, i.e. letting an algorithm make most critical decisions, much like directed evolution is an automated exploitation of natural selection. In the light of the recent successful designs using such protocols,[18–21] it is however sobering to note that each active structure is accompanied by a multitude of false positives.[22, 23] No reported *de novo* enzyme has significantly outperformed the numerous catalytic antibodies elicited in recent decades,[21, 22, 24] let alone wild-type analogs. Moreover, it has recently been reported that some *de novo* designs do not tolerate closer scrutiny[25] and that established protocols sometimes fail altogether.[26]

This mini review will focus on recent trends in improving *de novo* designs beyond the automation protocols, discussed within the framework of contemporary understanding of how enzymes work.[27–31] It does not cover all methodologies and results published recently; for this purpose, the reader is directed to other recent reviews[2, 23, 32–35] (also see ref. 2 and references therein).

## 2. Automated protein design

**Figure 1:**
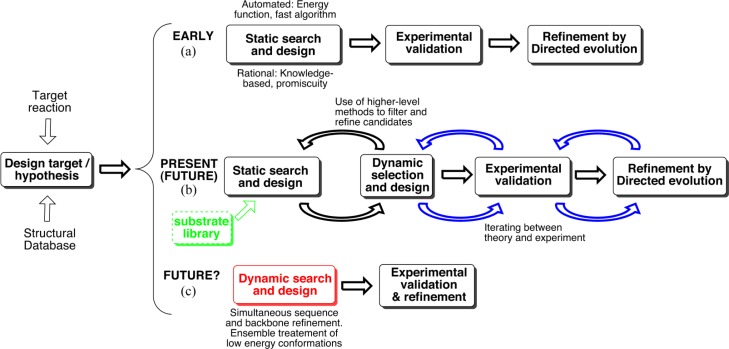
Evolution of enzyme design strategies. Nascent applications are indicated in blue and green and unlikely (or very distant) in red. (a) Approximate protocol based on automated and/or rational design followed by experimental validation and screening. Optionally, the designs can be further refined through directed evolution. (b) An iterative approach including molecular dynamics for selection and further design. (c) Tentative, automated computational design incorporating conformational flexibility and dynamics in the search algorithm.

Due to the sheer size of the sequence and conformational space of proteins, no computational method is able to assess them exhaustively. For example, the number of unique configurations when only considering a typical active site is ∼10^65^.[11] Hence, computational design relies on the ability to i) understand where to focus the efforts and ii) develop methods that efficiently examines and selects among the 'right’ variables. To this end, all automated design protocols so far start with an existing protein scaffold and redesigns its sequence around some introduced functionality while maintaining a rigid backbone (Figure 1a). We will refer to such approaches as 'static’. Protocols from the Mayo and Hellinga labs pioneered automated design, [3, 4, 36–38] and the first computational 'designer enzyme’ appeared in the early 2000's.[39 It was followed by an array of (re)designed binding proteins and enzymes.[8, 18, 38, 40] Although several reports from the Hellinga lab have been challenged[25] and in some cases retracted,[41] the numerous reports of de novo functions promised a bright future for computational enzyme design.

One of the most successful approaches has been to couple the structure prediction utilities in the Rosetta design package,[9, 42] developed by Kuhlman, Baker and coworkers,[10, 14, 43] with an 'inside-out’ active site-design initiated by a 'theozyme’[44] optimization. Three very impressive enzyme designs were published rapidly (the ‘Rosetta enzymes’), catalyzing a retro-aldol reaction,[19] a Kemp elimination,[20] and most recently a Diels-Alder reaction.[21] The inside-out design method is a clean-cut example of the general philosophy described above. It optimizes a minimal model involving the transition state (TS) and a few key interacting residues using quantum chemical (QC) methods, and the resulting theozyme is then fitted into a suitable active site, which is repacked with the Rosetta program. The idea relies on the finding that native proteins have nearly optimal sequences for their structure,[43] so that accommodating a new function can be done in a self-consistent way.

However, an enzyme needs to be exquisitely tuned in every part, not just the active site residues, to be proficient or even active.[45] As examplified in Table 1, enzymes straight out of the computer are typically quite weak catalysts. But fine-tuning distant residues is (so far) a bit to diffuse for computational methods,[45] and coupling with directed evolution has proven to be a more successful approach.[20, 46] Nevertheless, such refined enzymes have at best had a factor ∼10^2^ added to their effciencies (Table 1).

**Table 1:** Examples rate enhancements and specificities of computationally designed enzymes.^a^

Name	Reaction	$\log\frac{k_{\mathrm{cat}}}{k_{\mathrm{uncat}}}$	$\log\frac{k_{\mathrm{cat}}}{K_{M}}$	→log *K_TS_*	Ref.
P7D2	Ester hydrolysis	2.26	0.43	6.03	39
G_4_-DF_*tet*_	Phenol oxidation	≈3^b^	1.42	6.08	18
RA61	Retro-Aldol	4.36	-0.15	8.04	19
KE07	Kemp Elimination	4.19	1.11	7.04	20
→KE07*^c^	Kemp Elimination	6.07	3.40	9.34	20
KE70	Kemp Elimination	5.08	2.10	8.04	46
→KE70*^c^	Kemp Elimination	6.63	4.75	10.7	46
HG-3^d^	Kemp Elimination	5.77	2.63	8.56	79
DA_20_00	Diels-Alder^e^	0.61	-1.26	3.90	21
→DA_20_10^f^	Diels-Alder^e^	1.93	0.73	5.90	21,83
→CE6	Diels-Alder^e^	1.96	1.94	7.11	83

a A ’ →’ indicates the design has been developed from the closest design above.

b Only an approximate rate enhancement was reported by the authors.

c The 'R7 10/11G’ variant of KE07,[20] and 'R6 4/8B’ variant of KE70,[46] evolved by directed evolution and containing 8 and 14 mutations compared to their respective progenitor.

d Refined in three generations using a combination of small-molecule placement,[11] MD and experimental techniques.

e A bimolecular reaction, reported values therefore contain *k*_cat_/(*K*_*M1*_*K*_*M2*_).

f Evolved from DA_20_00 by rational design and contains 6 mutations with respect to the progenitor

g Evolved from DA_20_10 by exchanging an unstructured loop on the fringe of the active site to a helix-turn-helix motif that better encapsulates the substrates. The design was found by employing the community of FoldIt[16] players.

Before proceeding with a discussion on how to improve prediction and refinement of catalytic power in computational design, we will now briefly review how enzyme catalysis is understood. The framework will then be used to quantify substrate binding and dynamic contributions to the catalytic effect.

## 3. The underlying theory

In the famous words of Linus Pauling,[47, 48] enzymes work by ”stabilization of the transition state” of the reaction (relative to the solvent). Transition state theory (TST) has acquired additional flavors since then,[49] but it is now generally agreed that the bulk of the catalytic effect can be attributed to the quasi-thermodynamic transition state (TS) stabilization.[28] This is good news for the computational enzyme designer, since the 'extra-thermodynamic’[28] terms (re-crossing, tunneling and non-equilibrium effects) are arguably too subtle to be incorporated in computational predictions (yet).

**Figure 2:**
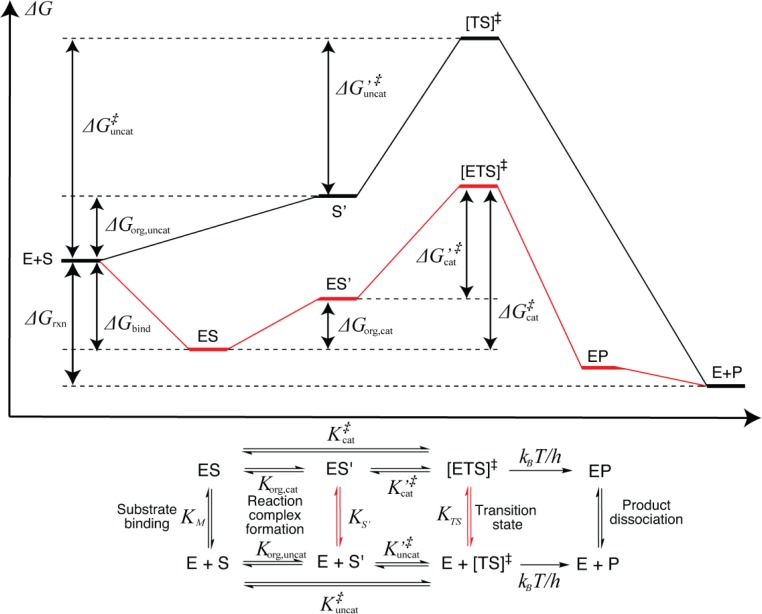
Schematic description of general enzyme catalysis (represented by one substrate and only the rate-determining step). The thermodynamic relationships in the bottom panel has been adapted from Wolfenden.[27] Fictitious equilibria are indicated in red.

TS stabilization is understood as higher affinity for the TS of the enzyme with respect to the solvent, described by a 'dissociation constant’ *K*_*TS*_.[50] This hypothetical TS binding is illustrated in the pseudo-thermodynamic cycle in Figure 2 and is defined as

$$K_{TS}=K_{M}\frac{K_{\mathrm{uncat}}^{‡}}{K_{\mathrm{cat}}^{‡}}=K_{M}\frac{k_{\mathrm{uncat}}}{k_{\mathrm{cat}}}$$

with the associated dissociation energy

$$\Delta \Delta G_{TS}=-RT\;\mathrm{In}\;K_{TS}=\Delta G_{\mathrm{uncat}}^{‡}-\Delta G_{\mathrm{cat}}^{‡}-\Delta G_{\mathrm{bind}}$$

(We allow ourselves to approximate *K*_*M*_ with exp[*▵G*_bind_*/RT*].) The catalytic proficiency of enzymes is thus the reciprocal of *K*_*TS*_ and can in native enzymes be as high as 10^24^ M^-1^. [51]

Exactly how enzymes achieve their TS stabilization has been, and still is, widely debated.[31, 52–54] That enzymes selectively bind their substrates is well understood[31] and reflected in small values of the Michaelis constant (*K*_*M*_). Rate enhancement, defined as *k*_cat_/*k*_uncat_, requires a lower enzymatic reaction barrier (*▵G*^‡^_cat_ < *▵G*^‡^_uncat_); if the difference is zero, *K*_*TS*_ simply equals *K*_*M*_. One way or another, a proficient enzyme must therefore allow a reaction path for which *k*_cat_*/k*_uncat_ >> 1, equivalent to *▵▵G*^‡^_cat_ = *▵G*^‡^_cat_ - *▵G*^‡^_uncat_ << 0.

The discourse regarding the origins of TS stabilization can, in simple terms, be said to be about whether they have completely pre-organized active sites that are evolved to match the TS geometry and electrostatics,[31, 55] or if they, e.g. by ground state destabilization (or entropy trapping),[56] desolvation[57] or dynamic motion,[30] increase the fraction of pre-organized states in conformational space.[53, 54] While it is reasonable to suggest that all contributions play a role,[58] it is still disputed which one dominates in a particular enzyme.[30, 59–61]

Regardless of what explanation one prefers, it is instructive for the present discussion to define a pre-organized state (S’ or ES’) as any substrate conformation that is distorted with respect to the ground state (S or ES), so that it lies 'en route’ to the TS on the reaction coordinate (Figure 2). Note that ES is a Boltzmann ensemble and contains all conformations defined to belong to ES’; the crucial delimiter between the two ensembles can be seen as ES not necessarily being conformationally similar to the TS while ES’ is. Thus, *▵G*_org_ > 0, and

$$\Delta G^{‡}=\Delta G_{\mathrm{org}}+\Delta {G^{\prime }}^{‡}$$

where *▵G*′^‡^ is the free energy of activation from the pre-organized state. We can from Figure 2 define a 'pre-organization constant’ *K*_*S′*_ as

$$K_{S^{\prime }}=K_{M}\frac{K_{\mathrm{org},\mathrm{uncat}}}{K_{\mathrm{org},\mathrm{cat}}}$$

and it follows that

$$K_{TS}=K_{{S^{\prime }}^{\exp}}\left[\frac{\Delta {G^{\prime }}_{\mathrm{cat}}^{‡}-\Delta {G^{\prime }}_{\mathrm{uncat}}^{‡}}{RT}\right]=K_{S^{\prime }}{K^{\prime }}_{TS}$$

This manipulation effectively divides the activation barrier in two parts, where *▵G*_org_ can be assumed to contain mainly non-electrostatic and entropic contributions, whereas *▵G′*^‡^ mainly contains the charge-transfer associated with the chemical step.[62]

From an experimental perspective it may seem pointless to consider such a ‘micro-history’[28, 63] of the reaction coordinate, since the only observables are *K*_*M*_ and *k*_cat_, but it is a useful computational tool to characterize the enzyme. And, as will be argued below, to design better ones. The introduction of an intermediate state does not dismiss any of the various theories regarding the origins of enzyme catalysis. Indeed, a very proficient native enzyme can be regarded as having an ES’ ES due to a pre-organized active site, in which case *▵G*_org,cat_ ≈ 0[60] and the extended model reduces to the usual one. That is, *▵G*^‡^_cat_ ≃ *▵G* ′^‡^_cat_ and contains solely electrostatic contributions[31] while all pre-organization effects are captured in *▵G*_bind_.

However, in designer enzymes the match might not be so ideal, even with the appropriate catalytic groups in place. Such a situation will render a larger *▵G*_org,cat_, and *k*_cat_/*k*_uncat_ will be small even with seemingly ideal interactions in the TS. In this case, the enzyme might be improved by 'pulling down’ ES’ towards ES, thereby lowering the barrier, whereas in a structure with insufficient electrostatic pre-organization, the effort must focus on placement of the catalytic residues to lower [ETS]^‡^.

## 4. Where automated design fails

How do these mechanistic considerations influence computational design strategies? Since the most direct design approach is to optimize an active site around a computed TS model or perhaps TS hybrid, the designer enzyme should ideally be fairly good at binding the substrate(s) in their pre-organized conformations (ES’) as well, with a consequently low *▵G*_org,cat_ penalty. In other words, optimizing TS binding should in principle yield adequate pre-arrangement as well. It has been pointed out that it is essentially the same strategy as employed when raising catalytic antibodies against haptens.[38]

As mentioned, such an approach essentially assumes that the design retains its structural integrity with respect to the scaffold, or at least that the region around the active site is rigid. Herein lies, the author argues, an important reason why computational design algorithms rarely exceed a rate enhancement of ∼10^6^ (Table 1),[22] and why the success rate is in fact very low.[21, 26] The dynamic nature of enzymes includes both backbone and side-chain movements, and although it is a good approximation to assume that the backbone remains rigid with respect to ∼10 introduced mutations, even a small distortion can severely affect the positioning of catalytic residues. A related problem is that backbone movements are slow on the molecular time scale, and they are therefore seldom captured in standard MD simulations.

Several studies have investigated the Rosetta enzymes,[46, 64–67] and most of their conclusions point in the same direction: Unaccounted protein dynamics leads to a unsatisfying description of enzyme-substrate interactions and the ability to pre-organize into the target TS, which results in an over-optimistic prediction of activity. In other words, the static nature of the design process fails to predict the actual Boltzmann distribution of ES conformations. In addition, Warshel and coworkers have pointed out that the extensively studied Kemp Eliminases attain a significant part of their catalytic effect by bringing the substrate close to the catalytic base, whereas actual TS stabilization is poor and extremely difficult to achieve due to a small difference in charge distribution compared to the ground state.[66]

It should be noted that the automated computational protocols do a fairly good job at predicting the (albeit simple) reaction mechanism and stereoselectivity, but do not manage very well to separate actives from inactives.[19–21] Thus, selection is a major challenge for automated protein design.[22] The problem of picking out a few structures from an immense ensemble is in principle equivalent to what troubles other disciplines in computational chemistry: calculating small energy differences between large systems.

## 5. Searching for matching functionalities

In order to mitigate some of the problems associated with introducing a *de novo* mechanism in an existing scaffold, an alternative strategy could be to search for structures with functionalities matching the desired machinery already in place. We have been interested in re-designing enzymes containing an 'oxyanion hole’ moiety[68] to catalyze the intermolecular Diels-Alder reaction, which is virtually unknown in nature[69–71] and therefore highly interesting for *de novo* design.[2] Our work was initially guided by a rational design framework (Figure 1a),[72] which can be said to rely on the concept of 'catalytic promiscuity’.[73] Catalytic promiscuity is in turn a manifest of evolutionary heritage,[23, 74, 75] where a wide array of reactions can be catalyzed by structurally and functionally similar enzymes.[23]

We recently adapted a 'semi-rational’ approach,[76, 77] which contains a biased search in the Protein Data Bank (PDB) for structures containing the target functionality (an oxyanion hole). The search is coupled with a search of a combinatorial substituent library for matching substrates, thereby increasing the chance of structural complementarity with the active site while keeping the number of mutations at a minimum (Figure 1b, green). After a pre-selection, the remaining structures are evaluated by molecular docking of TS analogs. The results then form the basis for further selection and rational mutations. Recently, Nosrati and Houk published a program ('SABER’) designed to automatically search the PDB for 'predesigns’ having a set of predefined functionalities in place for promiscuous catalysis.[78] Their approach is consistent with ours, albeit in a more automated fashion. To the author's knowledge, only the originnal proof of principle study has been published to date. One can anticipate, however, future *de novo* designs employing the SABER methodology.

## 6. Including dynamics

Another key feature of our approach is that we rely on molecular dynamics (MD) simulations for evaluation, selection and quantification of different variants,[72, 76] a practice that has recently begun appearing in several other studies (Figure 1b).[21, 67, 79] We refer to this as ‘dynamic design’.

The virtue of dynamic design is that it relaxes a static design in response to the functionalities introduced, and in addition provides both qualitative and quantitative tools to distinguish between systems with just a few atoms discrepancy (even though it does not warrant a perfect description of reality). It was shown by Kiss *et al*. that MD-derived metrics could be used to significantly improve computational predictions of active designs[67] of *de novo* Kemp Eliminases.[20] The groups of Mayo, Houk and Hilvert then went on to use MD in an iterative protocol[11] to refine an inactive design for the same reaction to an active one (HG-3 in Table 1).[79]

Despite the use of both QC and MD methods in several dynamic protocols, few attempts have been made so far to use them for quantitative predictions. Warshel and coworkers recently used their 'empirical valence bond’ (EVB) method to correlate computed *▵G*^‡^_cat_ values with experiment, and argued that this approach can be used for screening[80] and refinement purposes.[45] It is the author's opinion that reliable energetic predictions of the designed reaction coordinate is the most robust way to improve the predictive power of computational enzyme design.

We took an early interest in probing the possibility of quantitative evaluation using MD and QC. One reason for this being that the docking protocol used in the early stages of design is inherently static, like most other protocols employed thus far, and does not provide a particularly precise basis for selection of catalytic activity. Our studies have lead to a dynamic protocol employing standard MD and cluster QC calculations,[76, 77] which for a preliminary benchmark test gives encouraging results.

To quantify the quality of a particular design, one needs to determine *K*_*TS*_ (eq. 1), which requires explicit predictions of the substrate binding and activation free energies. An estimate of *K*_*M*_ can be obtained from the MD trajectory while the rate constants in principle require *ab initio* calculations. As seen from Figure 2, *▵G*^‡^_cat_ must be calculated with respect to ES, but this state is not correctly sampled at the QC level if not conformationally very restricted. Although QM/MM may be one solution to this problem, we have utilized that ES’ can be fairly well defined within both MD and QC frameworks due to its conformational constraints, and use eq. 3 to estimate *▵G*^‡^_cat_. The pre-organized complex can be defined in several ways, and we have used the concept of 'near attack conformers’ (NACs) championed by Bruice[81, 53] to determine ▵*G*_org,cat._ [60]

$$\Delta G_{\mathrm{org},\mathrm{cat}}≃\Delta G_{\mathrm{NAC}}=RT\mathrm{In}\frac{N_{\mathrm{NAC}}}{N_{\mathrm{total}}}$$

where *N*_NAC_ denotes the number of instances in the ensemble obeying the criteria defined for a NAC. Hence, we compute *▵G* ′^‡^ cat using a cluster model of the active site, and obtain ▵*G*_org,cat_ as a statistical average from MD. The uncatalyzed states are determined in a traditional way from QC.[72, 76, 77]

The NAC concept is debated[28, 54, 60, 61] and suffers from its inherently arbitrary definition,[28] but we argue that it is a useful tool to optimize substrate binding in enzyme catalysis. In the notation of Figure 2, NAC≡ES’ (or S’), and as pointed out above, the ideal design goal is to equate this state with ES. Computational design typically produces multiple candidates with essentially identical catalytic residues, and in a QC evaluation of the TS stabilization it is convenient to use the same (or similar) models to save CPU time. They can instead be ranked by augmenting a common *▵G* ′^‡^_cat_ with ▵*G*_org,cat_ obtained from MD. (This is essentially what is done by Houk and coworkers,[46, 67] although without estimating energetics.)

In addition, this treatment is useful for quantifying the origins of catalysis (or lack thereof!). If ES’ and S’ are defined equivalently, the electrostatic portion of the TS can be determined by *▵G* ′^‡^_cat_-*▵G* ′^‡^_uncat_ (*i.e*. *K′*_*TS*_ in eq. 5; this comparison is similar to the 'caged’ reference state used by Frushicheva *et al*.[45, 66]), which can be taken as a measure of the quality of the designed catalytic machinery.

As a test of the predictive power of this concept, the kinetic constants of four active Diels-Alderases published by the Baker group were estimated in ref. 77. We used a 'LIE+γSASA’ method to calculate binding constants from MD, and employed the theozyme reported by Siegel *et al*. as our cluster model. Despite its crudity, the approach predicted *K*_*TS*_ within one order of magnitude and ranked three of four designs correctly; the largest error was a factor 60 overestimation for CE6 (≈ 2.4 kcal·mol^-1^). Predicted in the same way, two variants designed using our semi-rational protocol have estimated rate enhancements of 5.9·10^4^ and 4.1·10^6^ M, and efficiencies of 8.6·10^4^ and 9.5·10^4^ s^-1^M^-1^, respectively.[76, 77] We have not yet been able to express these designs for experimental validation, but speculatively, our simulations suggest that the main difference between these variants and those published by the Baker group is the propensity to form NACs (or ES’). In our designs, we explicitly designed for alignment of the (ternary) ES complex to resemble the tentative TS, thereby lowering the *▵G*_org,cat_ penalty. We recorded *N*_NAC_/*N*_total_ values of ≤ 0.10 for the Baker variants, versus 0.25–0.50 for our best designs.

Interestingly, the computed *▵G*′^‡^ cat are not so different in these examples. In other words, we conclude that *K*′_*TS*_ is rather similar, whereas *K*_S′_ is smaller in our computational designs. This observation suggests that the moderate rate enhancements of the Diels-Alderases given in Table 1 can be improved by dedicated improvement of substrate pre-arrangement. In addition, the treatment reveals the difficulty in attaining a large (electrostatic) TS stabilization. We have that *▵G*′^‡^_uncat_ - ▵G′^‡^_cat_ ≤ 3 kcal/mol in our design studies. This can possibly be attributed to lack of electrostatic reorganization in the TS, similar to the case of Kemp-eliminases.[66]

Again, note that partitioning *K*_*TS*_ as in eq. 5 is not always justified, and must be done with care depending on what one seeks to analyze. We have used it as a tool to improve designs suffering from poor substrate pre-arrangement and quantitatively compare systems with similar active sites. Furthermore, the QC model needs to be large enough to capture all essential electrostatics, and the theozyme used for evaluating the Baker Diels-Alderases is, strictly speaking, too small.[45] Nevertheless, we obtained remarkable correlation with experiment. In our design works, we use cluster models of the active site for the QC calculations, which are typically size-converged at 150-200 atoms.[82]

## 7. Outlook

Ideally, one would wish for a completely dynamic design approach, where everything is treated in a time-resolved fashion (Figure 1c). For example, one would be able to measure the direct response to a point mutation or change in conformation of individual residues in 'real time’. Some methodologies have begun incorporating backbone flexibility,[13, 14, 32] and the EVB approach suggested by Warshel and coworkers[45, 66, 80] in principle maps the whole reaction coordinate. However, completely dynamic treatments will need time to mature, if ever feasible. A common and resilient problem is of course the coarseness of force field approaches, introducing dependencies on different parameterizations and failing to treat more subtle aspects of chemical interactions.

More likely is consolidation of the trend for which publications from the last couple of years[21, 46, 67, 78, 79, 83] provide mounting evidence: development of composite approaches that utilize rational, computational and experimental tools iteratively. Iteration between models and experiment has recently been demonstrated (Figure 1b, blue),[79] and additional protocols are likely to emerge. An intriguing example of the beneficial use of human resources is an enhanced Diels-Alderase design developed with the help of FoldIt players.[83] They collectively improved substrate binding of DA_20_10 by redesigning a loop surrounding the active site. The new design CE6 showed a 20-fold increased efficiency (see Table 1).

It has been argued that *K*^-1^_TS_ values larger than 10^11^ M^-1^ (▵▵*G*_*TS*_ ≈ 15 kcal·mol^-1^) are virtually impossible for enzymes exhibiting non-covalent mechanisms.[29] Non-covalent mechanisms have been defined as those involving covalent enzyme-substrate intermediates, general acid/base catalysis, metal coordination or low-barrier hydrogen bonds. It follows from Table 1 that all designs leave room for significant improvement. To accomplish this, enzyme designers must be prepared to envision more complex reactions.

Another pertinent aspect is that enzymes earn much of their proficiency from catalyzing reactions that are astoundingly slow in water,[27, 52] and for which catalysis stabilize large charge reorganizations in the TS.[84] Several of the reactions discussed herein are comparatively fast in solution, and thus limit the maximum rate enhancement. To significantly improve Diels-Alder catalysis, for example, it is perhaps necessary to both find a slower background reaction and re-route the catalytic mechanism. We recently presented an acid/base-mediated mechanism that utilized the catalytic machinery of ketosteroid isomerase.[85] This preliminary study indicated a dramatic rate enhancement of a reaction that is very slow in solution, provided that the substrates could bind to the active site and form pre-arranged conformations.

Enzyme design is evidently a complex, non-linear process and requires more than an ever-so-elegant algorithm. More advanced, diverse and cheap design tools, both computational and experimental, become available every year. The literature discussed in this review testifies that if a systematic application of the entire toolbox is conducted, dramatically improved results will definitely ensue.
